# Development of a multi-locus sequence typing scheme for *Laribacter hongkongensis*, a novel bacterium associated with freshwater fish-borne gastroenteritis and traveler's diarrhea

**DOI:** 10.1186/1471-2180-9-21

**Published:** 2009-01-30

**Authors:** Patrick CY Woo, Jade LL Teng, Alan KL Tsang, Herman Tse, Vivien YM Tsang, King-Man Chan, Edwin KY Lee, Jim KH Chan, Shirley SL Ma, Dorothy MW Tam, Liliane MW Chung, Susanna KP Lau, Kwok-Yung Yuen

**Affiliations:** 1 State Key Laboratory of Emerging Infectious Diseases, The University of Hong Kong, Hong Kong, PR China; 2 Research Centre of Infection and Immunology, The University of Hong Kong, Hong Kong, PR China; 3 Department of Microbiology, The University of Hong Kong, Hong Kong, PR China

## Abstract

**Background:**

Laribacter hongkongensis is a newly discovered, facultative anaerobic, Gram-negative, motile, sea gull-shaped rod associated with freshwater fish borne gastroenteritis and traveler's diarrhea. A highly reproducible and discriminative typing system is essential for better understanding of the epidemiology of *L. hongkongensis*. In this study, a multilocus sequence typing (MLST) system was developed for *L. hongkongensis*. The system was used to characterize 146 *L. hongkongensis *isolates, including 39 from humans and 107 from fish.

**Results:**

Fragments (362 to 504 bp) of seven housekeeping genes were amplified and sequenced. Among the 3068 bp of the seven loci, 332 polymorphic sites were observed. The median number of alleles at each locus was 34 [range 22 (*ilvC*) to 45 (*thiC*)]. All seven genes showed very low *d*_*n*_/*d*_*s *_ratios of < 0.04, indicating that no strong positive selective pressure is present. A total of 97 different sequence types (STs) were assigned to the 146 isolates, with 80 STs identified only once. The overall discriminatory power was 0.9861. eBURST grouped the isolates into 12 lineages, with six groups containing only isolates from fish and three groups only isolates from humans. Standardized index of association (*I*^*S*^_*A*_) measurement showed significant linkage disequilibrium in isolates from both humans and fish. The *I*^*S*^_*A *_for the isolates from humans and fish were 0.270 and 0.636, indicating the isolates from fish were more clonal than the isolates from humans. Only one interconnected network (*acnB*) was detected in the split graphs. The P-value (P = 0) of sum of the squares of condensed fragments in Sawyer's test showed evidence of intragenic recombination in the *rho, acnB *and *thiC *loci, but the P-value (P = 1) of maximum condensed fragment in these gene loci did not show evidence of intragenic recombination. Congruence analysis showed that all the pairwise comparisons of the 7 MLST loci were incongruent, indicating that recombination played a substantial role in the evolution of *L. hongkongensis*. A website for *L. hongkongensis *MLST was set up and can be accessed at http://mlstdb.hku.hk:14206/MLST_index.html.

**Conclusion:**

A highly reproducible and discriminative MLST system was developed for *L. hongkongensis*.

## Background

Laribacter hongkongensis is a newly discovered, facultative anaerobic, Gram-negative, motile, sea gull-shaped rod that belongs to the *Neisseriaceae *family of β-proteobacteria. It was first recovered from the blood and empyema thoracis of a patient with alcoholic liver cirrhosis in Hong Kong in 2001 [1]. Since the patient's underlying disease and the presence of ascites suggested that the gastrointestinal tract may be a possible source of infection, *L. hongkongensis *was intensively sought in human fecal specimens. During a period of two months, the bacterium was recovered from the stool of three patients with community-acquired gastroenteritis on charcoal cefoperazone deoxycholate agar. A similar finding was observed in three other patients in Switzerland [2]. Subsequently, in a multi-centered prospective study using a newly developed selective medium [3], the bacterium was shown to be associated with community-acquired gastroenteritis and traveler's diarrhea [4]. *L. hongkongensis *is likely to be globally distributed, as travel histories from patients suggested that it is present in at least four continents, including Asia, Europe, Africa and Central America [3,4]. Recently, *L. hongkongensis *has also been reported from another coastal province in mainland China [5]. In a recent review, *L. hongkongensis*, together with enterotoxigenic *Bacteroides fragilis *and *Klebsiella oxytoca*, were included as newly appreciated agents associated with acute diarrhea [6]. Although the causative role of *L. hongkongensis *in gastroenteritis is yet to be established [7], these data provide strong evidence that the bacterium is a potential diarrheal pathogen that warrants further investigations.

*L. hongkongensis *has been found in the intestines of healthy freshwater fish but not other studied animals that are commonly used for cooking in Hong Kong [4,8,9]. The bacterium was recovered from the guts of 24% of 360 freshwater fish studied, with the highest recovery rates from grass carp (60%) and bighead carp (53%) and during spring and summer [6,7]. Moreover, *L. hongkongensis *has also been recovered from drinking water reservoirs in Hong Kong [10]. The presence of a heterogeneous population of *L. hongkongensis *by pulsed-field gel electrophoresis (PFGE) among isolates from freshwater fish [9] and the association of *L. hongkongensis *gastroenteritis with fish consumption [4] suggested that freshwater fish is likely the major reservoir of the bacterium and the source of human infections.

A highly reproducible and discriminative typing system is essential for better understanding of the epidemiology of *L. hongkongensis*. Previously, we have used PFGE for typing *L. hongkongensis *[4,7,8]. However, due to experimental variations, PFGE patterns are difficult to compare among different laboratories. As multi-locus sequence typing (MLST) is well known to be highly reproducible and discriminative for bacteria, we developed such a typing system for *L. hongkongensis *using the sequence information of the *L. hongkongensis *complete genome sequence project. In this article, we report the development of an MLST scheme for *L. hongkongensis *using 146 isolates from humans and fish.

## Methods

### *L. hongkongensis *isolates

A total of 146 *L. hongkongensis *isolates, including 39 isolates recovered from humans and 107 isolates from fish, were used in this study (Additional file 1). *L. hongkongensis *isolate HKU1 was recovered from the blood culture and empyema pus of a patient with bacteremic empyema thoracis [1]. The other 38 isolates from humans (isolates HLHK2 to HLHK39) were recovered from the stool of patients with community-acquired gastroenteritis [2,4]. Isolates HLHK 2–4 were recovered from patients in Switzerland while the rest were from patients in Hong Kong. The 107 isolates from fish were recovered from the guts of freshwater fish sampled from retail food markets in Hong Kong [4,9]. These included 50 isolates (FLHK1–8, FLHK25–26, FLHK36–43, FLHK50–59, FLHK61–71, FLHK77–84 and FLHK94–96) recovered from grass carp (*Ctenoharyngodon idellus*), 42 isolates (FLHK9–14, FLHK27–33, FLHK44–49, FLHK72–76, FLHK85–93, FLHK97–100 and FLHK103–107) from bighead carp(*Aristichthys nobilis*), 12 isolates (FLHK15–21, FLHK34–35, FLHK60 and FLHK101–102) from mud carp (*Cirrhina molitorella*) and three isolates (FLHK22–24) from large-mouth bass(*Micropterus salmoides*). The identification of all *L. hongkongensis *isolates were confirmed phenotypically by standard conventional biochemical methods and genotypically by 16S rRNA gene sequencing [1,4].

### DNA extraction

Bacterial DNA extraction was modified from our previous published protocol [1]. Briefly, 800 μl of NaOH (0.05 M) was added to 200 μl of bacterial cells suspended in distilled water and the mixture was incubated at 60°C for 45 min, followed by addition of 240 μl of Tris-HCl (pH 7.0), achieving a final pH of 8.0. The resultant mixture was diluted 100× and 0.5 μl of the diluted extract was used for PCR.

### PCR amplification and sequencing

Extracted DNA from the 146 isolates of *L. hongkongensis *was used as the template for amplification of seven housekeeping genes [transcription termination facter Rho (*rho*); aconitate hydratase (*acnB*); cell division protein (*ftsH*); anthranilate synthase component I (*trpE*); ketol-acid reductoisomerase (*ilvC*); thiamin biosynthesis protein ThiC (*thiC*); enolase (*eno*)], using primers listed in Table 1. The seven housekeeping genes were chosen because either the gene itself or other genes in the same metabolic pathway has been used in MLST schemes of other bacteria. The sequences of the seven genes were obtained from our on-going *L. hongkongensis *complete genome sequence project (unpublished data).

**Table 1:** Primers for amplification and sequencing of the seven housekeeping genes in *L. hongkongensis*

Gene locus	Primers		Amplicon size (bp)
		
	Forward	Reverse	
*rho*	LPW868 5'-ATCGTCCTNNTGATTGACGAG-3'	LPW869 5'-GATGTTGATGGCCNGGAA-3'	468
*acnB*	LPW870 5'-GGCANGCCTTCGATTTCG-3'	LPW871 5'-GCGNCCGGGNACNTACTG-3'	561
*ftsH*	LPW872 5'-CGGTCGAAACGGCCNGGG-3'	LPW873 5'-CGGNTGNGACGAAGCCAA-3'	480
*trpE*	LPW1712 5'-ACGGNGACATCATGCAGG-3'	LPW1713 5'-NACNGCNGTGCGGATGGC-3'	626
*ilvC*	LPW1714 5'-GCNGCCAACGGCGGCACCAA-3'	LPW1715 5'-AAGGCATCATNGCNCGCAG-3'	473
*thiC*	LPW1716 5'-ATCATGGCCAANTGGTGTCT-3'	LPW1717 5'-GCCTTGNNNAGCGCGTTGTC-3'	557
*eno*	LPW3137 5'-CGCTGCGGGGCGGAAATCTT-3'	LPW2555 5'-CCAGATCAACGGCCTTGAGC-3'	517

The PCR mixture (100 μl) contained denatured *L. hongkongensis *DNA, PCR buffer (10 mM Tris-HCl pH 8.3 and 50 mM KCl), 2 mM MgCl_2_, 200 μM of each deoxynucleoside triphosphates and 2.5 U Ampli *Taq *Gold DNA polymerase (Applied Biosystems, Foster City, CA, USA). For *rho*, *trpE*, *ilvC*, *thiC *and *eno*, the sample was amplified in 40 cycles of 94°C for 1 min, 55°C for 1.5 min and 72°C for 2 min, and with a final extension at 72°C for 10 min in an automated thermal cycler (Applied Biosystems, Foster City, CA, USA). For *acnB *and *ftsH*, the sample was amplified using a reannealing temperature of 60°C. Twenty microliters of each amplified product was electrophoresed in 2% (w/v) agarose gel, with a molecular size marker (GeneRuler™ 50 bp DNA ladder, MBI Fermentas, Canada). Electrophoresis in Tris-borate-EDTA buffer was performed at 120 volts for 40 min. The gel was stained with ethidium bromide (0.5 μg/ml) for 15 min, rinsed and photographed under ultraviolet light illumination.

The PCR product was gel-purified using the QIAquick PCR purification kit (QIAgen, Hilden, Germany). Both strands of the PCR product were sequenced using BigDye Terminator Cycle Sequencing kit version 3.1 with an ABI Prism 3700 DNA Analyzer according to manufacturers' instructions (Applied Biosystems, Foster City, CA, USA) and the PCR primers. BioEdit version 7.0.5.2 was used for reading the sequences and aligning the forward and backward reads [11].

### Allele and sequence type assignment

The nucleotide sequences of the seven gene loci used for MLST in all the *L. hongkongensis *isolates were aligned and compared with those of isolate HLHK1 using Clustal W multiple alignment [12] implemented in BioEdit version 7.0.5.2 [11]. An arbitrary number was assigned to each distinct allele at a locus. The numbered alleles at each locus were combined in order to establish the sequence type (ST) for each isolate. Each ST was numbered in the order of identification (ST-1, ST-2, etc.). The data have been deposited in our *Laribacter hongkongensis *complete genome sequence and MLST database http://mlstdb.hku.hk:14206/MLST_index.html

### Sequence analysis

The proportions of nucleotide alterations that led to a change in the amino acid sequence (non-synonymous substitution, *d*_*n*_) and the proportions of nucleotide alterations that did not lead to a change in the amino acid sequence (synonymous substitution, *d*_*s*_) were calculated with START2 http://pubmlst.org/software/analysis/[13]. Phylogenetic analysis was performed using ClonalFrame algorithm with the software package ClonalFrame version 1.1, using 50,000 burn-in cycles and 100,000 further iterations [14]. Over 500 trees were generated from which a 75% majority-rule consensus tree was derived with MEGA version 4.0 [15]. STs were grouped into lineages with eBURST [16]. The members of an eBURST lineage were defined as groups of two or more independent isolates where each had identical alleles at six or more loci with at least one other member of the group. The linkage disequilibrium between alleles at the seven gene loci was measured using the standardized index of association (*I*^*S*^_*A*_) with LIAN 3.5 http://pubmlst.org/analysis/[17,18]. Split decomposition analysis was performed using the SplitsTree program (version 4.10) [19]. Sawyer's test analysis for intragenic recombination was performed with START2 http://pubmlst.org/software/analysis/[13]. Gene tree congruence analysis was performed using the Shimodaira-Hasegawa (SH) test [20] as implemented in PAUP 4.0b10 using the RELL method and 10000 bootstrap replicates [21]. Ninety-seven STs were selected and used in the SH test. Maximum-likelihood trees for each MLST gene of the 97 STs were inferred under a general time-reversible model, with an estimated gamma distribution, using PHYML v3.0 [22].

## Results

### Variation at the seven MLST loci

Single bands of the expected sizes were observed for each gene locus amplified using the specific primers. Among the 3068 bp of the seven loci, a total of 332 polymorphic sites were observed in the 146 isolates of *L. hongkongensis*. Two hundred and sixty-five and 246 polymorphic sites were observed in the 39 isolates from humans and 107 isolates from fish respectively. No insertion, deletion or premature termination was observed in any of the polymorphic sites. Allelic profiles were assigned to the 146 isolates of *L. hongkongensis *(Additional file 1). The alleles defined for the MLST system were based on sequence lengths of between 362 bp (*ilvC*) and 504 bp (*acnB*). The median number of alleles at each locus was 34 [range 22 (*ilvC*) to 45 (*thiC*)].

The *d*_*n*_/*d*_*s *_ratio for the seven gene loci are shown in Table 2. All seven genes showed very low *d*_*n*_/*d*_*s *_ratios of < 0.04 (median 0.0154, range 0.0000 – 0.0355), indicating that no strong positive selective pressure is present.

**Table 2:** Characteristics of loci and Sawyer's test analysis for intragenic recombination in *L. hongkongensis *isolates

Locus	Size of sequenced fragment (bp)	No. of alleles identified	No. (%) of polymorphic nucleotide sites	% G + C	*d*_*n*_/*d*_*s*_	SSCF^a ^(P-value)^b^	MCF^c ^(P-value)
*rho*	399	31	40 (10.0%)	58.7%	0.0000	160937 (0)*	39 (1)
*acnB*	504	39	45 (8.9%)	66.6%	0.0043	281863 (0)*	43 (1)
*ftsH*	428	43	46 (10.7%)	63.4%	0.0126	392301 (0.53)	43 (1)
*trpE*	448	34	44 (9.8%)	59.4%	0.0265	174730 (0.46)	37 (1)
*ilvC*	362	22	16 (4.4%)	58.3%	0.0154	11688 (0.55)	14 (1)
*thiC*	473	45	101 (21.4%)	63.3%	0.0355	954286 (0)*	92 (1)
*eno*	454	31	40 (8.8%)	60.5%	0.0266	118330 (0.18)	33 (1)

^a^SSCF, sum of the squares of condensed fragments

^b^P-value indicating statistically significant (P < 0.05) evidence for recombination are marked with asterisks

^c^MCF, maximum condensed fragment

### Relatedness of *L. hongkongensis *isolates

**Figure 1:**
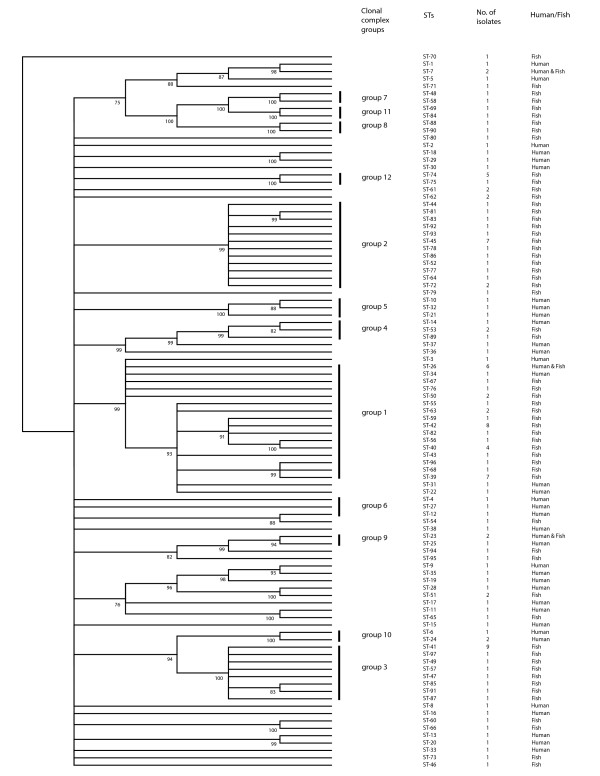
**Phylogenetic tree showing the relationships of the 97 STs of *L. hongkongensis *in this study**. The genetic relatedness among the 97 STs was assessed by ClonalFrame algorithm based on the pair-wise differences in the allelic profiles of the seven housekeeping genes. Numbers immediately to the right of the dendrogram show the eBURST clonal clusters to which the STs belong.

A total of 97 different STs were assigned to the 146 *L. hongkongensis *isolates, with 80 of the 97 STs identified only once (Additional file 1). Thirty-eight different STs were observed in the 39 isolates from humans and 62 different STs observed in the 107 isolates from fish, with 29 STs in 50 grass carp, 30 STs in 42 bighead carps, 11 STs in the 12 mud carps and two STs in three large mouth bass. Three STs (ST-7, ST-23 and ST-26) were found in both isolates from humans and fish. The most common ST (ST-41) was identified nine times, followed by ST-42 (eight isolates) and ST-45 (seven isolates). The overall discriminatory power for the 146 isolates was 0.9861, that for the isolates from 39 humans was 0.9987 and for the isolates from fish was 0.9755. ClonalFrame was used to construct a dendrogram using the concatenated nucleotide sequences of the seven gene loci of the 146 isolates (Fig. 1).

**Figure 2:**
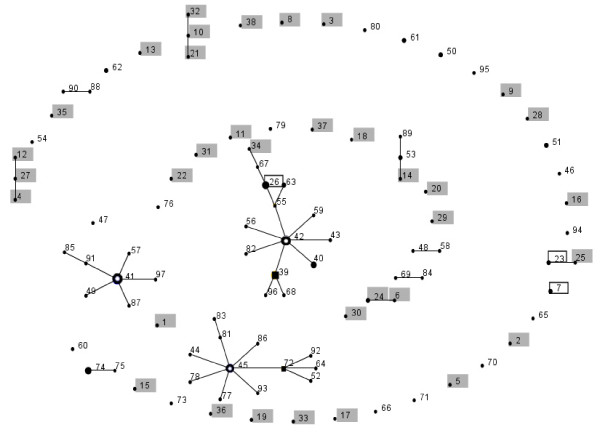
**BURST analysis of *L. hongkongensis *isolates in this study**. Each number represents a MLST sequence type (ST) and each line connects STs that differ in only one of the seven housekeeping genes. Boxed numbers represent STs found in both human and fish, shaded numbers represent STs found only in human, and un-boxed and un-shaded numbers represent STs found only in fish. Hollow circles and squares represent predicted group and subgroup founders respectively. The sizes of the circles and squares are proportional to the number of isolates within each ST.

**Figure 3:**
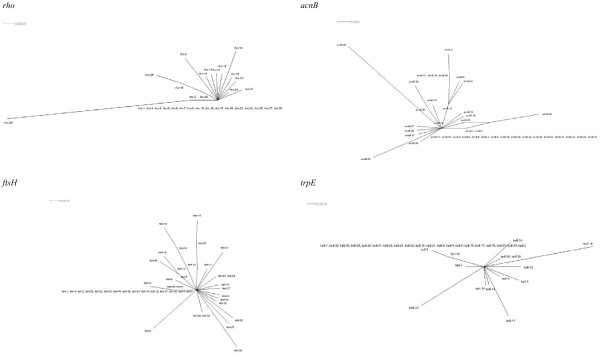
**Split decomposition analysis of MLST data of *L. hongkongensis *isolates in this study**. Split decomposition network was constructed using the individual (*rho*, *acnB, ftsH, trpE*, *ilvC*, *thiC *and *eno*) gene sequences. The scale bar represents the number of substitutions per site.

eBURST grouped the isolates into 12 lineages, with 14 STs in group 1, 12 STs in group 2, seven STs in group 3, three STs in groups 4–6 and two STs in groups 7–12, whereas 43 STs did not belong to any of the 12 groups (Fig. 2 and Additional files 1 and 2). These 43 singleton STs were isolated from 25 patients and 19 fish (one ST was found in both). All these 12 groups were also observed as clusters in the dendrogram (Fig. 1). Groups 2, 3, 7, 8, 11 and 12 contained only isolates from fish, group 1 contained 34 isolates from fish and two isolates from humans, group 4 contained three isolates from fish and one isolate from human, group 9 contained one isolate from fish and two isolates from humans, and groups 5, 6 and 10 contained only isolates from human. *I*^*S*^_*A *_measurement showed significant linkage disequilibrium in both isolates from humans and fish. The *I*^*S*^_*A *_for the isolates from humans and fish were 0.270 (0.243 if the three isolates from Switzerland were removed and 0.251 if the allelic profiles of the 38 unique STs of the isolates from humans were used) and 0.636 (0.469 if the allelic profiles of the 59 unique STs of the isolates from fish were used), indicating that the isolates from fish were more clonal than the isolates from humans. Only one interconnected network (*acnB*) was detected in the split graphs (Fig. 3). The P-value (P = 0) of sum of the squares of condensed fragments in Sawyer's test showed evidence of intragenic recombination in the *rho, acnB *and *thiC *loci, but the P-value (P = 1) of maximum condensed fragment in these gene loci did not show evidence of intragenic recombination (Table 2). Congruence analysis showed that all the pairwise comparisons of the 7 MLST loci were incongruent, indicating that recombination played a substantial role in the evolution of *L. hongkongensis*. (Table 3).

**Table 3:** Shimodaira-Hasegawa test for congruency among tree topologies for the seven loci and their concatenated sequence^a^

Locus	Results							
	
	Concatenation	*rho*	*acnB*	*ftsH*	*trpE*	*ilvC*	*thiC*	*eno*
Concatenation		0.0000*	0.0000*	0.0000*	0.0000*	0.0000*	0.0000*	0.0000*
*rho*	0.0001*		0.0000*	0.0000*	0.0001*	0.0000*	0.0000*	0.0000*
*acnB*	0.0001*	0.0000*		0.0000*	0.0000*	0.0000*	0.0000*	0.0000*
*ftsH*	0.0003*	0.0002*	0.0002*		0.0003*	0.0002*	0.0002*	0.0003*
*trpE*	0.0001*	0.0000*	0.0001*	0.0000*		0.0000*	0.0000*	0.0000*
*ilvC*	0.0075*	0.0090*	0.0064*	0.0048*	0.0056*		0.0059*	0.0072*
*thiC*	0.0000*	0.0000*	0.0000*	0.0000*	0.0000*	0.0000*		0.0000*
*eno*	0.0008*	0.0003*	0.0008*	0.0003*	0.0008*	0.0008*	0.0008*	

^a^P values (*, P < 0.05) represent differences in likelihood score between the maximum likelihood topology of each locus

No relationships were observed among the *L. hongkongensis *isolates with respect to their years of isolation; the locations of the hospitals, age and sex of the patients and the presence of plasmids in the isolates from patients [23]; nor to the species of the fish and the locations of the markets where the fish were purchased.

## Discussion

A highly discriminative MLST scheme was developed for *L. hongkongensis*. Seven housekeeping genes with very low *d*_*n*_/*d*_*s *_ratios of the range of 0.0000 – 0.0355, similar to the housekeeping genes in other MLST schemes, were employed to produce a highly discriminative MLST scheme, with discriminatory power of 0.9861, comparable to the MLST schemes of other pathogenic bacteria, for molecular typing of *L. hongkongensis*. When the same *L. hongkongensis *isolate was subcultured 50 times, no difference was observed between the sequences of the seven gene loci in the original isolate and the one after 50 subcultures (data not shown). Therefore, these seven loci are discriminative enough for typing, but not evolving too rapidly to an extent that will mask genetic relatedness, as in the case of *Helicobacter pylori*, another urease positive, S-shaped and motile alimentary tract microbe [24,25].

The *L. hongkongensis *isolates recovered from fish were clustered. In our previous study on ecoepidemiology of *L. hongkongensis *using PFGE of *Spe*I digested chromosomal DNA, it was found that the isolates from fish and humans were clustered separately [9]. Analysis of the sequences of the seven gene loci using both dendrogram and eBURST groups revealed a similar phenomenon to the previous ecoepidemiology study, although the clustering pattern of the isolates in the present study was different from that in the previous one (data not shown). eBURST group analysis showed that six of the 12 groups consisted exclusively of isolates from fish, whereas three of the 12 groups consisted exclusively of isolates from humans (Fig. 2). All these 12 eBURST groups were also found in clusters in the dendrogram (Fig. 1), although *I*^*S*^_*A *_measurement showed that the isolates from fish were probably more clonal than the isolates from humans. All these results of clustering of isolates from fish and humans into different groups observed in both the previous PFGE and the present MLST studies suggested that some clones of *L. hongkongensis *could be more virulent than others.

Although the isolates from fish appeared more clonal than the isolates from humans, a heterogeneous population of *L. hongkongensis *existed in the same ecosystem. STs recovered from the same species of fish or the same fish market did not cluster together. Over 80% of freshwater fish consumed in Hong Kong are imported from fish farms in mainland China, whereas the remaining 20% are locally reared in fish farms in rural areas of Hong Kong. Since the same species of freshwater fish in a particular market is usually obtained from the same fish farm and multiple STs were present in *L. hongkongensis *isolates recovered from the same species purchased from the same market, it implied that multiple clones of *L. hongkongensis *probably existed in the same aquaculture farm in mainland China or Hong Kong.

## Conclusion

Seven housekeeping genes with very low *d*_*n*_/*d*_*s *_ratios were employed to produce a highly discriminative MLST scheme for molecular typing of *L. hongkongensis*.

## Abbreviations

bp: base pairs; *d*_*n*_: non-synonymous substitution; *d*_*s*_: synonymous substitution; DNA: deoxyribonucleic acid; *I*^*S*^_*A*_: standardized index of association; M: molar; min: minutes; ml: milliliter; MLST: multilocus sequence typing; PCR: polymerase chain reaction; PFGE: pulsed-field gel electrophoresis; ST: sequence type; μl: microliter.

## Authors' contributions

PCYW conceived the study and drafted the manuscript. PCYW, JLLT, SKPL and KYY participated in the design of the study. PCYW and JLLT supervised the study. PCYW, JLLT and AKLT analyzed the data. HT constructed the database and website. KMC, EKYL, JKHC, SSLM, DMWT and LMWC carried out the PCR and sequencing experiments. SKPL and KYY corrected the manuscript. All authors read and approved the final manuscript.

## Supplementary Material

Additional file 1 **Characteristics of *L. hongkongensis *isolates used in the present study.** The tabulated data describe the background epidemiological and MLST characteristics of the 146 *L. hongkongensis *isolates in this study. Click here for file

Additional file 2 **eBURST groups of *L. hongkongensis *isolates.** The tabulated data provide the detailed compositions of each eBURST group of *L. hongkongensis *isolates. Click here for file
