# Supplementary material for: Development of a multi-locus sequence typing scheme for Laribacter hongkongensis, a novel bacterium associated with freshwater fish-borne gastroenteritis and traveler's diarrhea
Source: BMC Microbiol. 2009 Jan 30;9:21. doi: 10.1186/1471-2180-9-21 (PMC2644701; doi:10.1186/1471-2180-9-21)
Supplement: Additional file 1 — Characteristics of L. hongkongensis isolates used in the present study. The tabulated data describe the background epidemiological and MLST characteristics of the 146 L. hongkongensis isolates in this study. [file 1471-2180-9-21-S1.doc]

| Isolate | Year of isolation | Origin | Hospital/  Market^a,b^ | Sex/Age^a^ | ST | eBURST group^c^ | Allelic profile |
| --- | --- | --- | --- | --- | --- | --- | --- |
| HLHK1 | 1995 | Human | A | M/54 | ST-1 | Singleton | 1,1,1,1,1,1,1 |
| HLHK2 | 1998 | Human | NA | M/1 | ST-2 | Singleton | 2,2,2,2,2,2,2 |
| HLHK3 | 1999 | Human | NA | F/29 | ST-3 | Singleton | 3,3,3,3,3,3,3 |
| HLHK4 | 1999 | Human | NA | F/21 | ST-4 | 6 | 4,4,4,4,4,4,4 |
| HLHK5 | 2002 | Human | B | M/51 | ST-5 | Singleton | 1,5,5,5,5,5,5 |
| HLHK6 | 2002 | Human | A | M/63 | ST-6 | 10 | 5,6,6,6,6,6,6 |
| HLHK7 | 2002 | Human | B | M/35 | ST-7 | Singleton | 1,7,1,1,1,7,7 |
| HLHK8 | 2002 | Human | B | F/82 | ST-8 | Singleton | 6,8,7,7,7,8,8 |
| HLHK9 | 2002 | Human | C | F/36 | ST-9 | Singleton | 7,9,7,8,8,9,7 |
| HLHK10 | 2002 | Human | D | M/60 | ST-10 | 5* | 8,10,8,9,9,10,9 |
| HLHK11 | 2002 | Human | D | M/1 | ST-11 | Singleton | 9,11,9,10,10,11,10 |
| HLHK12 | 2002 | Human | B | F/20 | ST-12 | 6 | 4,12,10,4,4,4,4 |
| HLHK13 | 2002 | Human | A | F/41 | ST-13 | Singleton | 10,13,9,11,9,12,11 |
| HLHK14 | 2002 | Human | A | F/34 | ST-14 | 4 | 7,9,11,12,8,13,12 |
| HLHK15 | 2002 | Human | A | F/74 | ST-15 | Singleton | 11,14,6,13,11,14,13 |
| HLHK16 | 2002 | Human | B | F/89 | ST-16 | Singleton | 12,15,1,14,9,15,11 |
| HLHK17 | 2002 | Human | B | M/58 | ST-17 | Singleton | 10,16,1,15,9,16,14 |
| HLHK18 | 2002 | Human | B | M/45 | ST-18 | Singleton | 13,17,12,16,12,17,11 |
| HLHK19 | 2002 | Human | B | M/30 | ST-19 | Singleton | 7,9,13,17,9,18,15 |
| HLHK20 | 2002 | Human | D | M/20 | ST-20 | Singleton | 14,18,12,18,13,19,11 |
| HLHK21 | 2002 | Human | C | F/57 | ST-21 | 5 | 8,10,8,9,9,10,8 |
| HLHK22 | 2002 | Human | C | M/50 | ST-22 | Singleton | 3,19,3,19,1,3,16 |
| HLHK23 | 2002 | Human | E | M/9m | ST-23 | 9 | 15,20,7,20,8,20,16 |
| HLHK24 | 2002 | Human | B | M/41 | ST-24 | 10 | 5,6,6,6,9,6,6 |
| HLHK25 | 2003 | Human | D | F/28 | ST-25 | 9 | 15,20,14,20,8,20,16 |
| HLHK26 | 2003 | Human | D | M/30 | ST-26 | 1 | 3,3,3,19,14,3,15 |
| HLHK27 | 2003 | Human | C | M/56 | ST-27 | 6* | 4,12,4,4,4,4,4 |
| HLHK28 | 2003 | Human | B | F/21 | ST-24 | 10 | 5,6,6,6,9,6,6 |
| HLHK29 | 2003 | Human | C | F/71 | ST-28 | Singleton | 16,20,15,10,9,18,12 |
| HLHK30 | 2003 | Human | C | M/84 | ST-29 | Singleton | 17,21,16,21,15,21,12 |
| HLHK31 | 2003 | Human | B | M/9m | ST-30 | Singleton | 18,22,17,22,9,22,17 |
| HLHK32 | 2003 | Human | C | F/32 | ST-31 | Singleton | 3,19,18,19,1,3,4 |
| HLHK33 | 2003 | Human | D | M/46 | ST-32 | 5 | 8,10,8,9,16,10,9 |
| HLHK34 | 2003 | Human | A | F/86 | ST-33 | Singleton | 5,18,19,18,17,23,18 |
| HLHK35 | 2003 | Human | D | F/81 | ST-34 | 1 | 3,3,3,23,18,3,15 |
| HLHK36 | 2003 | Human | B | M/77 | ST-35 | Singleton | 19,9,6,24,11,24,11 |
| HLHK37 | 2003 | Human | B | F/53 | ST-36 | Singleton | 16,23,20,17,9,25,19 |
| HLHK38 | 2003 | Human | B | F/69 | ST-37 | Singleton | 20,9,13,17,9,25,12 |
| HLHK39 | 2003 | Human | B | M/35 | ST-38 | Singleton | 21,12,4,4,8,26,15 |
| FLHK1 | 2002 | Grass carp | F | NA | ST-39 | 1 | 3,19,21,19,14,3,15 |
| FLHK2 | 2002 | Grass carp | G | NA | ST-40 | 1 | 3,19,19,19,14,19,15 |
| FLHK3 | 2002 | Grass carp | H | NA | ST-41 | 3* | 5,24,22,24,19,27,20 |
| FLHK4 | 2002 | Grass carp | I | NA | ST-42 | 1* | 3,19,19,19,14,3,15 |
| FLHK5 | 2002 | Grass carp | J | NA | ST-42 | 1* | 3,19,19,19,14,3,15 |
| FLHK6 | 2002 | Grass carp | K | NA | ST-43 | 1 | 3,19,7,19,14,3,15 |
| FLHK7 | 2002 | Grass carp | L | NA | ST-42 | 1* | 3,19,19,19,14,3,15 |
| FLHK8 | 2002 | Grass carp | L | NA | ST-44 | 2 | 22,25,3,25,9,28,21 |
| FLHK9 | 2002 | Bighead carp | F | NA | ST-40 | 1 | 3,19,19,19,14,19,15 |
| FLHK10 | 2002 | Bighead carp | G | NA | ST-45 | 2* | 22,25,23,25,9,28,21 |
| FLHK11 | 2002 | Bighead carp | M | NA | ST-26 | 1 | 3,3,3,19,14,3,15 |
| FLHK12 | 2002 | Bighead carp | N | NA | ST-46 | Singleton | 10,26,24,6,20,23,11 |
| FLHK13 | 2002 | Bighead carp | J | NA | ST-47 | Singleton | 22,24,19,24,19,27,20 |
| FLHK14 | 2002 | Bighead carp | L | NA | ST-26 | 1 | 3,3,3,19,14,3,15 |
| FLHK15 | 2002 | Mud carp | F | NA | ST-48 | 7 | 23,27,25,26,9,29,8 |
| FLHK16 | 2002 | Mud carp | F | NA | ST-41 | 3* | 5,24,22,24,19,27,20 |
| FLHK17 | 2002 | Mud carp | G | NA | ST-49 | 3 | 5,24,22,24,19,30,20 |
| FLHK18 | 2002 | Mud carp | I | NA | ST-7 | Singleton | 1,7,1,1,1,7,7 |
| FLHK19 | 2002 | Mud carp | I | NA | ST-50 | Singleton | 3,28,26,27,4,31,22 |
| FLHK20 | 2002 | Mud carp | M | NA | ST-51 | Singleton | 16,20,27,23,9,18,12 |
| FLHK21 | 2002 | Mud carp | N | NA | ST-52 | 2 | 24,25,23,25,21,28,21 |
| FLHK22 | 2002 | Large-mouth bass | M | NA | ST-53 | 4* | 7,9,11,12,8,25,12 |
| FLHK23 | 2002 | Large-mouth bass | N | NA | ST-53 | 4* | 7,9,11,12,8,25,12 |
| FLHK24 | 2002 | Large-mouth bass | L | NA | ST-54 | Singleton | 4,22,10,4,4,32,4 |
| FLHK25 | 2002 | Grass carp | F | NA | ST-55 | 1 | 3,19,3,19,14,3,15 |
| FLHK26 | 2002 | Grass carp | F | NA | ST-39 | 1 | 3,19,21,19,14,3,15 |
| FLHK27 | 2002 | Bighead carp | F | NA | ST-23 | 9 | 15,20,7,20,8,20,16 |
| FLHK28 | 2002 | Bighead carp | F | NA | ST-56 | 1 | 3,19,19,19,14,33,15 |
| FLHK29 | 2002 | Bighead carp | F | NA | ST-57 | 3 | 5,24,28,24,19,27,20 |
| FLHK30 | 2002 | Bighead carp | F | NA | ST-58 | 7 | 23,27,29,26,9,29,8 |
| FLHK31 | 2002 | Bighead carp | F | NA | ST-41 | 3* | 5,24,22,24,19,27,20 |
| FLHK32 | 2002 | Bighead carp | F | NA | ST-59 | 1 | 3,19,19,19,14,34,15 |
| FLHK33 | 2002 | Bighead carp | F | NA | ST-39 | 1 | 3,19,21,19,14,3,15 |
| FLHK34 | 2002 | Mud carp | F | NA | ST-45 | 2* | 22,25,23,25,9,28,21 |
| FLHK35 | 2002 | Mud carp | F | NA | ST-60 | Singleton | 25,29,30,28,22,35,23 |
| FLHK36 | 2002 | Grass carp | G | NA | ST-61 | Singleton | 20,9,19,29,9,36,11 |
| FLHK37 | 2002 | Grass carp | G | NA | ST-62 | Singleton | 26,9,31,30,9,37,20 |
| FLHK38 | 2002 | Grass carp | G | NA | ST-63 | 1 | 3,30,3,19,14,3,15 |
| FLHK39 | 2002 | Grass carp | G | NA | ST-41 | 3* | 5,24,22,24,19,27,20 |
| FLHK40 | 2002 | Grass carp | G | NA | ST-64 | 2 | 22,25,32,25,21,28,21 |
| FLHK41 | 2002 | Grass carp | G | NA | ST-63 | 1 | 3,30,3,19,14,3,15 |
| FLHK42 | 2002 | Grass carp | G | NA | ST-42 | 1* | 3,19,19,19,14,3,15 |
| FLHK43 | 2002 | Grass carp | G | NA | ST-65 | Singleton | 15,31,33,10,2,11,10 |
| FLHK44 | 2002 | Bighead carp | G | NA | ST-66 | Singleton | 25,29,34,28,21,38,24 |
| FLHK45 | 2002 | Bighead carp | G | NA | ST-42 | 1* | 3,19,19,19,14,3,15 |
| FLHK46 | 2002 | Bighead carp | G | NA | ST-67 | 1 | 3,3,3,23,14,3,15 |
| FLHK47 | 2002 | Bighead carp | G | NA | ST-68 | 1 | 3,19,21,19,14,34,15 |
| FLHK48 | 2002 | Bighead carp | G | NA | ST-69 | 11 | 27,32,25,26,9,29,8 |
| FLHK49 | 2002 | Bighead carp | G | NA | ST-70 | Singleton | 6,33,35,31,2,36,25 |
| FLHK50 | 2002 | Grass carp | H | NA | ST-45 | 2* | 22,25,23,25,9,28,21 |
| FLHK51 | 2002 | Grass carp | H | NA | ST-71 | Singleton | 1,34,36,17,2,39,8 |
| FLHK52 | 2002 | Grass carp | H | NA | ST-42 | 1* | 3,19,19,19,14,3,15 |
| FLHK53 | 2002 | Grass carp | H | NA | ST-72 | 2 | 22,25,23,25,21,28,21 |
| FLHK54 | 2002 | Grass carp | H | NA | ST-51 | Singleton | 16,20,27,23,9,18,12 |
| FLHK55 | 2002 | Grass carp | H | NA | ST-73 | Singleton | 28,35,37,32,9,23,26 |
| FLHK56 | 2002 | Grass carp | I | NA | ST-45 | 2* | 22,25,23,25,9,28,21 |
| FLHK57 | 2002 | Grass carp | I | NA | ST-61 | Singleton | 20,9,19,29,9,36,11 |
| FLHK58 | 2002 | Grass carp | I | NA | ST-41 | 3* | 5,24,22,24,19,27,20 |
| FLHK59 | 2002 | Grass carp | I | NA | ST-74 | 12 | 29,36,38,33,6,40,27 |
| FLHK60 | 2002 | Mud carp | I | NA | ST-50 | Singleton | 3,28,26,27,4,31,22 |
| FLHK61 | 2002 | Grass carp | M | NA | ST-39 | 1 | 3,19,21,19,14,3,15 |
| FLHK62 | 2002 | Grass carp | M | NA | ST-74 | 12 | 29,36,38,33,6,40,27 |
| FLHK63 | 2002 | Grass carp | M | NA | ST-75 | 12 | 29,36,38,33,6,41,27 |
| FLHK64 | 2002 | Grass carp | M | NA | ST-41 | 3* | 5,24,22,24,19,27,20 |
| FLHK65 | 2002 | Grass carp | M | NA | ST-74 | 12 | 29,36,38,33,6,40,27 |
| FLHK66 | 2002 | Grass carp | M | NA | ST-74 | 12 | 29,36,38,33,6,40,27 |
| FLHK67 | 2002 | Grass carp | M | NA | ST-41 | 3* | 5,24,22,24,19,27,20 |
| FLHK68 | 2002 | Grass carp | N | NA | ST-76 | Singleton | 3,3,17,23,14,42,15 |
| FLHK69 | 2002 | Grass carp | N | NA | ST-74 | 12 | 29,36,38,33,6,40,27 |
| FLHK70 | 2002 | Grass carp | N | NA | ST-77 | 2 | 3,25,23,25,9,28,21 |
| FLHK71 | 2002 | Grass carp | N | NA | ST-45 | 2* | 22,25,23,25,9,28,21 |
| FLHK72 | 2002 | Bighead carp | N | NA | ST-39 | 1 | 3,19,21,19,14,3,15 |
| FLHK73 | 2002 | Bighead carp | N | NA | ST-78 | 2 | 22,25,23,25,9,28,28 |
| FLHK74 | 2002 | Bighead carp | N | NA | ST-79 | Singleton | 30,23,9,17,9,28,29 |
| FLHK75 | 2002 | Bighead carp | N | NA | ST-26 | 1 | 3,3,3,19,14,3,15 |
| FLHK76 | 2002 | Bighead carp | N | NA | ST-39 | 1 | 3,19,21,19,14,3,15 |
| FLHK77 | 2002 | Grass carp | J | NA | ST-62 | Singleton | 26,9,31,30,9,37,20 |
| FLHK78 | 2002 | Grass carp | J | NA | ST-80 | Singleton | 31,37,39,9,2,43,8 |
| FLHK79 | 2002 | Grass carp | J | NA | ST-81 | 2 | 22,25,40,25,9,28,21 |
| FLHK80 | 2002 | Grass carp | J | NA | ST-82 | 1 | 3,19,19,19,9,3,15 |
| FLHK81 | 2002 | Grass carp | J | NA | ST-83 | 2 | 22,25,40,25,19,28,21 |
| FLHK82 | 2002 | Grass carp | J | NA | ST-41 | 3* | 5,24,22,24,19,27,20 |
| FLHK83 | 2002 | Grass carp | J | NA | ST-84 | 11 | 27,32,25,26,21,29,8 |
| FLHK84 | 2002 | Grass carp | J | NA | ST-85 | 3 | 5,38,23,24,19,27,20 |
| FLHK85 | 2002 | Bighead carp | J | NA | ST-86 | 2 | 22,25,23,25,19,28,21 |
| FLHK86 | 2002 | Bighead carp | J | NA | ST-87 | 3 | 5,24,41,24,19,27,20 |
| FLHK87 | 2002 | Bighead carp | J | NA | ST-88 | 8 | 27,39,31,26,9,44,8 |
| FLHK88 | 2002 | Bighead carp | J | NA | ST-89 | 4 | 7,9,41,12,8,25,12 |
| FLHK89 | 2002 | Bighead carp | J | NA | ST-90 | 8 | 27,39,23,26,9,44,8 |
| FLHK90 | 2002 | Bighead carp | J | NA | ST-72 | 2 | 22,25,23,25,21,28,21 |
| FLHK91 | 2002 | Bighead carp | J | NA | ST-41 | 3* | 5,24,22,24,19,27,20 |
| FLHK92 | 2002 | Bighead carp | J | NA | ST-91 | 3 | 5,24,23,24,19,27,20 |
| FLHK93 | 2002 | Bighead carp | J | NA | ST-92 | 2 | 22,25,22,25,21,28,21 |
| FLHK94 | 2002 | Grass carp | K | NA | ST-45 | 2* | 22,25,23,25,9,28,21 |
| FLHK95 | 2002 | Grass carp | K | NA | ST-93 | 2 | 22,25,42,25,9,28,21 |
| FLHK96 | 2002 | Grass carp | K | NA | ST-94 | Singleton | 15,20,23,20,8,9,16 |
| FLHK97 | 2002 | Bighead carp | K | NA | ST-95 | Singleton | 15,20,19,34,8,45,30 |
| FLHK98 | 2002 | Bighead carp | K | NA | ST-40 | 1 | 3,19,19,19,14,19,15 |
| FLHK99 | 2002 | Bighead carp | K | NA | ST-96 | 1 | 3,19,21,19,14,3,31 |
| FLHK100 | 2002 | Bighead carp | K | NA | ST-40 | 1 | 3,19,19,19,14,19,15 |
| FLHK101 | 2002 | Mud carp | K | NA | ST-97 | 3 | 5,24,43,24,19,27,20 |
| FLHK102 | 2002 | Mud carp | K | NA | ST-39 | 1 | 3,19,21,19,14,3,15 |
| FLHK103 | 2002 | Bighead carp | L | NA | ST-42 | 1* | 3,19,19,19,14,3,15 |
| FLHK104 | 2002 | Bighead carp | O | NA | ST-42 | 1* | 3,19,19,19,14,3,15 |
| FLHK105 | 2002 | Bighead carp | O | NA | ST-26 | 1 | 3,3,3,19,14,3,15 |
| FLHK106 | 2002 | Bighead carp | O | NA | ST-45 | 2* | 22,25,23,25,9,28,21 |
| FLHK107 | 2002 | Bighead carp | O | NA | ST-26 | 1 | 3,3,3,19,14,3,15 |

^a^NA, not applicable.

^b^Hospital/Market indicates the hospital that the patient visited or the market where the fish was purchased. Isolates HLHK2-4 were recovered from patients in Switzerland [2].

^c^Ancestral type in eBURST analysis is marked with an asterisk.
