# Supplementary material for: Development of a multi-locus sequence typing scheme for Laribacter hongkongensis, a novel bacterium associated with freshwater fish-borne gastroenteritis and traveler's diarrhea
Source: BMC Microbiol. 2009 Jan 30;9:21. doi: 10.1186/1471-2180-9-21 (PMC2644701; doi:10.1186/1471-2180-9-21)
Supplement: Additional file 2 — eBURST groups of L. hongkongensis isolates. The tabulated data provide the detailed compositions of each eBURST group of L. hongkongensis isolates. [file 1471-2180-9-21-S2.doc]

| eBURST group | STs^a^ | No. of ST | No. of human isolates | No. of fish isolates | No. of isolates | Isolates |
| --- | --- | --- | --- | --- | --- | --- |
| 1 | 26, 34, 39, 40, 42*, 43, 55, 56, 59, 63, 67, 68, 82 , 96 | 14 | 2 | 34 | 36 | FLHK105, FLHK107, FLHK11, FLHK14, FLHK75, HLHK26, HLHK35, FLHK1, FLHK102, FLHK26, FLHK33, FLHK61, FLHK72, FLHK76, FLHK100, FLHK2, FLHK9, FLHK98, FLHK103, FLHK104, FLHK4, FLHK42, FLHK45, FLHK5, FLHK52, FLHK7, FLHK6, FLHK25, FLHK28, FLHK32, FLHK38, FLHK41, FLHK46, FLHK47, FLHK80, FLHK99 |
| 2 | 44, 45*, 52, 64, 72, 77, 78, 81, 83, 86, 92, 93 | 12 | 0 | 19 | 19 | FLHK8, FLHK10, FLHK106, FLHK34, FLHK50, FLHK56, FLHK71, FLHK94, FLHK21, FLHK40, FLHK53, FLHK90, FLHK70, FLHK73, FLHK79, FLHK81, FLHK85, FLHK93, FLHK95 |
| 3 | 41*, 49, 57, 85, 87, 91, 97 | 7 | 0 | 15 | 15 | FLHK16, FLHK3, FLHK31, FLHK39, FLHK58, FLHK64, FLHK67, FLHK82, FLHK91, FLHK17, FLHK29, FLHK84, FLHK86, FLHK92, FLHK101 |
| 4 | 14, 53*, 89 | 3 | 1 | 3 | 4 | HLHK14, FLHK22, FLHK23, FLHK88 |
| 5 | 10*, 21, 32 | 3 | 3 | 0 | 3 | HLHK10, HLHK21 , HLHK33 |
| 6 | 4, 12, 27* | 3 | 3 | 0 | 3 | HLHK12, HLHK27, HLHK4 |
| 7 | 48, 58 | 2 | 0 | 2 | 2 | FLHK15, FLHK30 |
| 8 | 88, 90 | 2 | 0 | 2 | 2 | FLHK87, FLHK89 |
| 9 | 23, 25 | 2 | 2 | 1 | 3 | FLHK27, HLHK23, HLHK25 |
| 10 | 6, 24 | 2 | 3 | 0 | 3 | HLHK24, HLHK28, HLHK6 |
| 11 | 69, 84 | 2 | 0 | 2 | 2 | FLHK48, FLHK83 |
| 12 | 74, 75 | 2 | 0 | 6 | 6 | FLHK59, FLHK62, FLHK65, FLHK66, FLHK69, FLHK63 |

^a^Ancestral type in eBURST analysis is marked with an asterisk.
